# Endermic Method of Using Remedies

**Published:** 1829-04

**Authors:** 


### ENDERMIC METHOD OF USING REMEDIES.

*Articular Rheumatism.*—A young man, a mason, was admitted into the Hôpital Cochin with acute rheumatism of the joints, and a gastro-enteritis. In all his joints, but particularly in his arms, he had excruciating pains and stiffness, by which he was rendered incapable of motion. There were likewise frequent sweats, orbital headach, foul tongue, quick pulse, and constipation. By repeated bloodletting and leeches the pain was removed from the abdomen; but it continued without abatement elsewhere, and the accompanying fever assumed the adynamic form. The leechbites on the belly being carefully cleaned, half a grain of morphia was introduced into them, and with the effect of procuring him a quiet night's rest, and subsequent alleviation of all his pains and uneasiness. The morphia having been then intermitted, the pains returned severely. They were alleviated, however, by a blister on the right thigh; but soon recurred with equal violence, and continued so for eleven days. Half a grain of morphia was then applied to the blistered surface, and it was followed by complete absence of pain and refreshing sleep. Next day the morphia was given by mistake internally, and caused bilious vomiting, dry tongue, stupor, and somnolency. The rheumatic pains, however, continued to abate, and soon ceased altogether, without any further application of the remedy.

*Sciatica.*—A middle-aged man was suddenly seized with this complaint in the left haunch, and, after suffering severely for three weeks, got gradually better by wearing a Burgundy pitch plaster. But, after having been again at his work for nearly six weeks, he had a more severe attack than the former, by which he was compelled to have recourse to an hospital. The left limb was considerably emaciated, the knee rigidly bent, the whole movements of the limb accompanied with acute pain extending from the hip to the heel. In bed the pains were unceasing, and wholly prevented sleep. The toes at the same time were contracted involuntarily, the muscles affected with subsultus, the skin with a sense of creeping. Warm baths were of no avail. Two moxas applied near the chief seats of the pain caused full suppuration, with as little advantage. Half a grain of morphia was then applied to the suppurating surfaces; and in an hour the pains ceased, and the man fell asleep. The same treatment was continued for nearly six weeks with little intermission, the dose being first raised gradually to two grains, and then gradually diminished to one grain daily. At first every intermission, even for a single day only, was followed by a return of severe pain. But after six weeks, although the remedy was abandoned altogether, the disease gradually disappeared. On one occasion the morphia was given by mistake internally, and caused nausea, vomiting, stuttering, revery, tremors, dysuria, itching of the skin, succeeded by dulness, wildness of expression, and general discomfort.

*Lead Palsy.* Case I.—A lapidary was attacked with colica pictonum in March 1824, and was cured of it at La Charité. In January 1825, tremors having seized his arms, he returned to that hospital, and was again cured by baths and milk diet. In June he was seized with racking pains in his limbs and palsy of the hands, especially of the extensors of the fingers, which were quite powerless. Electro-puncture was tried with very little advantage, and subsequently friction with tincture of strychnia, without any relief. A blister was then applied on the outside of each forearm, and three days afterwards, while the paralysis was still as great as ever, half a grain of strychnia was sprinkled over the blistered surface on the right forearm. Next morning, the patient having in the interval slept soundly, he was greatly surprised to find that he could extend his right hand and fingers completely, while the left remained powerless as before. At night half a grain was sprinkled over each blistered surface, and next morning the left hand could be extended, and the right was stronger. This treatment was repeated several days, and the patient eventually recovered the former strength of his arms. On one occasion, a grain having been applied on each arm, contractions and vermicular movements of the muscles of the hand were immediately induced, and afterwards heat, sweating, general succussions, and formication.

Case II.—A red-lead manufacturer, who had been eighteen times cured of the lead colic at the hospital of La Charité, was attacked with a palsy of the extensors of the hands, during convalescence from his last attack of colic; and, after taking a few pills of strychnia, was dismissed as incurable. The whole movements of both hands, but particularly of the right hand, were sluggish and limited; but the extensors of the fingers and hands were quite powerless. A blister being applied to the outside of each forearm, half a grain of strychnia was sprinkled on the exposed surface of one. In an hour he felt a strong sensation of heat of skin and dragging in the muscles of the hand, and next morning he could extend the fingers a little. The treatment was then intermitted for a few days, on account of the recurrence of an habitual giddiness; but, on being resumed, a grain of the strychnia was applied on the blistered parts, and in an hour slight succussions took place in both arms, and indeed throughout the whole body. Next day the fingers of the right hand could be freely extended, and those of the left could be raised considerably. On the day after that, the powder was applied to the right arm, and caused contractions in that arm only. After another intermission, on account of a return of the vertigo, the treatment was again resumed, and for several days two grains were daily applied to an open blister on the nape of the neck. The hands, in consequence, were gradually and progressively restored to their natural state.

